# *CALM1*, *CALM2*, and *CALM3* expression and translation efficiency provide insight into the severity of calmodulinopathy

**DOI:** 10.1093/europace/euag052

**Published:** 2026-03-18

**Authors:** Steffan Noe Niikanoff Christiansen, Stine Bøttcher Jacobsen, Jeppe Dyrberg Andersen, Ya Cui, Wei Li, Christian Staehr, Mikkel Meyer Andersen, Lia Crotti, Carla Spazzolini, Peter J Schwartz, Mette Nyegaard, Michael Toft Overgaard

**Affiliations:** Department of Health Science and Technology, Aalborg University, Selma Lagerløfs Vej 249, 9260 Gistrup, Aalborg, Denmark; Department of Forensic Medicine, University of Copenhagen, Frederik V’s Vej 11, 2100 Copenhagen, Denmark; Department of Forensic Medicine, University of Copenhagen, Frederik V’s Vej 11, 2100 Copenhagen, Denmark; Department of Forensic Medicine, University of Copenhagen, Frederik V’s Vej 11, 2100 Copenhagen, Denmark; Division of Computational Biomedicine, Department of Biological Chemistry, University of California, Irvine, CA, USA; Division of Computational Biomedicine, Department of Biological Chemistry, University of California, Irvine, CA, USA; Department of Biomedicine, Health, Aarhus University, Aarhus, Denmark; Department of Anesthesiology and Intensive Care, Aarhus University Hospital, Aarhus, Denmark; Department of Forensic Medicine, University of Copenhagen, Frederik V’s Vej 11, 2100 Copenhagen, Denmark; Department of Mathematical Sciences, Aalborg University, Aalborg, Denmark; Center for Cardiac Arrhythmias of Genetic Origin and Laboratory of Cardiovascular Genetics, Istituto Auxologico Italiano IRCCS, Milano, Italy; Departments of Medicine and Surgery, University of Milano-Bicocca, Milan, Italy; Center for Cardiac Arrhythmias of Genetic Origin and Laboratory of Cardiovascular Genetics, Istituto Auxologico Italiano IRCCS, Milano, Italy; Center for Cardiac Arrhythmias of Genetic Origin and Laboratory of Cardiovascular Genetics, Istituto Auxologico Italiano IRCCS, Milano, Italy; Department of Health Science and Technology, Aalborg University, Selma Lagerløfs Vej 249, 9260 Gistrup, Aalborg, Denmark; Department for Congenital Disorders, Statens Serum Institut, Copenhagen, Denmark; Department of Chemistry and Bioscience, Aalborg University, Aalborg, Denmark

**Keywords:** Calmodulinopathy, Calmodulin, *CALM1*, *CALM2*, *CALM3*, Untranslated regions, Gene expression, Translational efficiency

## Abstract

**Aims:**

Missense variants in the *CALM1*, *CALM2*, and *CALM3* genes cause calmodulinopathy, which is characterized by ventricular arrhythmias and sudden cardiac death. Although the three genes encode an identical protein, their individual roles and gene-specific clinical implications remain poorly understood. We aimed to determine the relative contribution from each of the genes to the total calmodulin amount and assess the consequence of missense mutations on the severity of calmodulinopathy.

**Methods and results:**

Using data from the Genotype-Tissue Expression (GTEx) project, we show that *CALM2* constituted a higher percentage of the calmodulin-coding mRNA (41.9%) compared with *CALM1* (36.8%) and *CALM3* (21.3%) (*P* < 2 × 10^−16^). Paired RNA sequencing and ribosome profiling data from the left ventricle was used to demonstrate that the translation into calmodulin protein was significantly different among *CALM1* (44.8%) and *CALM2* (44.2%), and *CALM3* (11.0%) (*P* < 2 × 10^−16^). The observed-to-expected ratio for the number of missense variants in the Genome Aggregation Database (gnomAD) was 0.29 (90% CI, 0.23–0.36) in *CALM3*, 0.20 (90% CI, 0.15–0.27) in *CALM2*, and 0.11 in *CALM1* (90% CI, 0.07–0.17). In the International Calmodulinopathy Registry, a different percentage of carriers experiencing cardiac events was observed among those with missense variants in *CALM1* (46/52, 89%), *CALM2* (37/53, 70%), and *CALM3* (20/35, 57%) (*P* = 0.004).

**Conclusion:**

Compared with *CALM1* and *CALM2*, *CALM3* is under less negative selection and missense variant carriers are less prone to cardiac events. We suggest this is partially due to *CALM3* accounting for only 11% of the calmodulin protein produced in the ventricles.

## Graphical Abstract

**Graphical Abstract:**
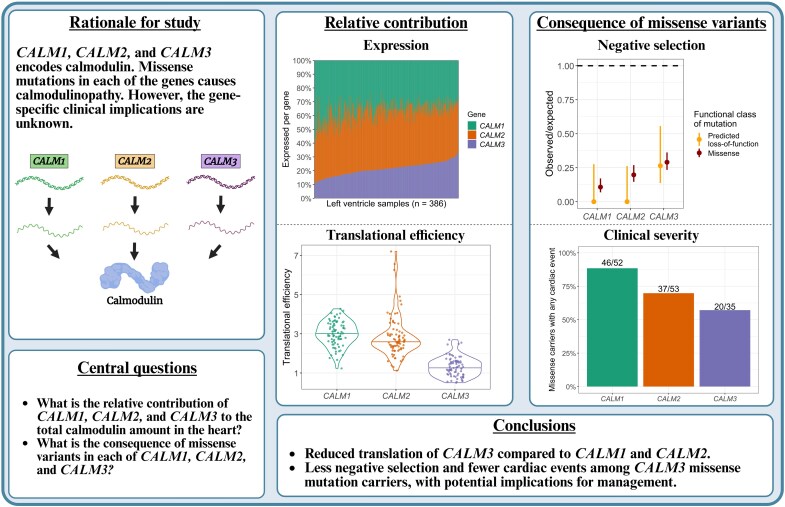

Translational perspectives Although *CALM1*, *CALM2*, and *CALM3* encode an identical calmodulin protein, the gene-specific expression and translation differ substantially, which is consistent with different frequencies of missense mutation carriers in clinical and population studies. These findings underscore the importance of gene-specific variant interpretation in calmodulinopathy. Moreover, understanding the distinct functional consequences of missense mutations in *CALM1*, *CALM2*, and *CALM3* is essential for advancing precision medicine approaches in calmodulinopathy.

## Introduction

DNA variants that change the protein sequence of any of the calmodulin-encoding genes (*CALM1*, *CALM2*, and *CALM3*) cause calmodulinopathy, an ultra-rare disorder associated with a broad spectrum of clinical manifestations.^1–3^ These symptoms include long QT syndrome (LQTS),^4^ catecholaminergic polymorphic ventricular tachycardia (CPVT),^5^ sudden cardiac death in childhood and adolescence, and neurodevelopmental features.^6,7^ While some missense variants are observed to follow a dominant inheritance pattern characterized by variable expressivity of symptoms among the carriers across multiple generations,^5,8^ approximately 80% of the index cases are assumed to carry *de novo* mutations.^7^

The *CALM1*, *CALM2*, and *CALM3* genes are highly conserved, with the calmodulin amino acid sequence being identical and evolutionarily conserved across all vertebrates.^9^ Depletion of a specific *CALM* gene harbouring a *CALM* missense variant can alleviate the arrhythmia characteristics in either cultured cardiomyocytes or mice by reducing the ratio between mutated and normal calmodulin.^10,11^ Yet, naturally occurring depletion by loss-of-function mutations in any of *CALM1*, *CALM2*, and *CALM3* are extremely rare suggesting that such mutations are removed by selection due to reduced survival or reproducibility.

The dosage of mutated calmodulin is linked to the arrhythmic characteristics in mice.^12^ Therefore, it is possible that the relative contribution of *CALM1*, *CALM2*, and *CALM3* to the total cardiac calmodulin amount affect the phenotypic severity in patients with calmodulinopathy. However, as the relative contribution cannot be measured directly on protein level due to sequence identity, the understanding of relative expression and translation among *CALM1*, *CALM2*, and *CALM3* is particularly important.

Therefore, we tested two hypotheses: (i) the relative expression and translational efficiency is different among *CALM1*, *CALM2*, and *CALM3* in the heart, and (ii) the consequence of missense variants is specific for each of *CALM1*, *CALM2*, and *CALM3*. To address hypothesis I, we compared the gene expression using data from the from the Genotype-Tissue Expression (GTEx) project.^13^ Furthermore, we assessed the translational activity of *CALM1*, *CALM2*, and *CALM3* in left ventricular tissue to estimate the relative contribution of each gene to the shared pool of calmodulin protein. To address hypothesis II, we compared the observed-to-expected ratios of variants in the three genes using population data from the Genome Aggregation Database (gnomAD),^14^ and we compared the frequency of cardiac events among *CALM1*, *CALM2*, and *CALM3* missense variant carriers in the International Calmodulinopathy Registry.^7^

## Methods

**Figure 1:**
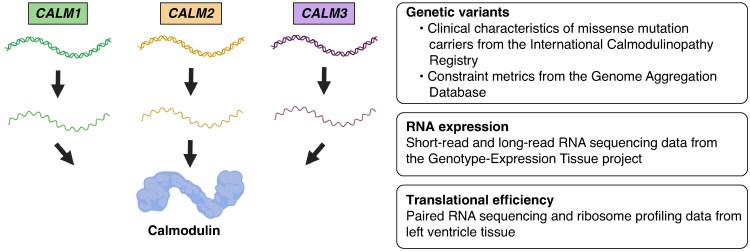
Design of study. *CALM1*, *CALM2*, and *CALM3* encode an identical calmodulin protein. To assess the relative contribution from *CALM1*, *CALM2*, and *CALM3* to the cardiac calmodulin amount, we investigated transcript and gene expression based on data from the Genotype-Expression Tissue (GTEx) project^13^ and translational efficiency based on paired RNA sequencing and ribosome profiling data.^18^ To determine the consequence of missense variants in each of the genes, we compared genetic constraint based on data from the Genome Aggregation Database (gnomAD)^14^ and clinical characteristics among missense variant carriers based on data from the International Calmodulinopathy Registry.^7^

The study was designed as outlined in *Figure 1*. The data underlying the findings of this study is available online (see Data availability).

### RNA-sequencing data from the genotype-tissue expression project

RNA sequencing data was obtained from the GTEx project, which is a database and public resource that has been established with the primary goal of studying gene expression among human tissues.^13^ The samples were collected from non-diseased tissues of donors aged 21 to 70 years.^15^

We used long-read RNA sequencing data obtained from GTEx v9^16^ to determine which *CALM1*, *CALM2*, and *CALM3* transcripts that were expressed in human tissues and to validate the gene expression of short-read RNA sequencing data. The short-read RNA sequencing data was obtained from GTEx v8^17^ and used to determine the relative gene expression among *CALM1*, *CALM2*, and *CALM3* in 49 tissues. The mapped read counts were generated according to the procedures described in the GTEx portal (https://gtexportal.org/home/methods). The transcript annotations were performed using the GENCODE (v26) annotation file.

### RNA-sequencing and ribosome profiling data from left ventricle tissue

RNA sequencing and ribosomal profiling data was obtained from a previous investigation of left ventricle tissue.^18^ In brief, the study included data from 22 female and 58 male subjects aged 1 to 77 years. The majority of the samples were obtained during surgical procedures of individuals affected by dilated cardiomyopathy (*n* = 65) while the remaining samples were obtained from unused donor hearts (*n* = 13), autopsy after trauma (*n* = 1), or from valve sparing aortic root replacement (*n* = 1). Ribosomal profiling enables investigation of the translating ribosomes by sequencing of the short mRNA fragments that are protected from nuclease degradation by the ribosomes.^19^ In the original processing of the data, the reads from the RNA sequencing and ribosomal profiling were trimmed to the same length and processed with the exact same settings.^18^

### *CALM1*, *CALM2*, and *CALM3* missense variants in the international calmodulinopathy registry and the genome aggregation database

We used data from two cohorts to investigate the potential selection pressure on variants in *CALM1*, *CALM2*, or *CALM3*. The International Calmodulinopathy Registry is a multicentre clinical observational registry that was established in 2015 to recruit patients carrying a pathogenic variant in *CALM1*, *CALM2*, or *CALM3* independent of their phenotype.^6^ The patients were mainly enrolled through centres and experts in arrhythmic disease. Clinical characteristics of 140 subjects with missense variants in *CALM1*, *CALM2*, or *CALM3* were obtained from the most recent version of the International Calmodulinopathy Registry.^7^ We compared any cardiac event corresponding to arrhythmic syncope, aborted cardiac arrest, sudden cardiac death, or appropriate implantable cardioverter–defibrillator shocks.

Observed-to-expected ratios and the accompanying 90% confidence intervals (CI) of missense variants and predicted loss-of-function variants were obtained from gnomAD, which is a collection of data from large-scale sequencing projects. gnomAD (v4.1) includes data from 730 947 exomes and 76 215 genomes. Predicted loss-of-function variants included stop-gain, frameshift, and splice-altering variants as previously defined.^20^ The expected number of mutations per gene was estimated based on a mutational model as previously described.^20^ The data was filtered based on the ‘mane_select’ variable to obtain a single representative transcript for each gene as previously described.^21^

### Data analysis

The data analysis was performed in the R statistical environment (version 4.4.1) using the tidyverse (version 2.0.0)^22^ and patchwork (version 1.3.0)^23^ packages. The genomic regions were visualized with the ggtranscript (version 0.99.9)^24^ package. The chi-squared tests were conducted with *chisq.test()*, Fisher’s Exact tests were conducted with *fisher.test()*, the Wilcoxon signed rank tests were conducted with *wilcox.test()*, and Spearman’s correlation coefficients, ρ, were calculated with *cor.test(method = ‘spearman’)*. For all the statistical tests, we accounted for multiple testing by only considering the results significant if the *P*-values were lower than the significance threshold, 0.05, divided by the number of tests.

### Estimation of 3′ untranslated region usage

To account for different usage of 3′-untranslated regions (UTR) among *CALM1*, *CALM2*, and *CALM3*, we calculated the percentage of distal poly(A) site usage index (PDUI) values for the GTEx short-read sequencing data as previously described.^25^ The approach was based on the DaPars v.2 framework.^26,27^ In brief, based on multiple RNA sequencing samples, the location of proximal alternative polyadenylation sites is estimated together with expression levels of the short and long 3′-UTRs. The output is the alternative polyadenylation usage reported per gene and sample.

### Gene expression

The data analysis of the GTEx short-read and long-read RNA sequencing data were based on either mapped read counts or the supplied transcript per million (TPM) values. The data were filtered to only include *CALM1*, *CALM2*, and *CALM3*.

To account for the different gene lengths of *CALM1*, *CALM2*, and *CALM3*, within-sample comparisons among the three genes was performed with read counts adjusted for gene lengths as shown below:


$$\mathrm{Counts}\;\mathrm{per}\;\mathrm{nt}=\frac{\mathrm{read}\;\mathrm{counts}}{\mathrm{total}\;\mathrm{transcript}\;\mathrm{length}}=\;\frac{\mathrm{read}\;\mathrm{counts}}{l_{5^{\prime }-\mathrm{UTR}}+\;l_{\mathrm{CDS}}+\;l_{3^{\prime }-\mathrm{UTR}}}$$

where *l*_5′-UTR_, *l_-_*_CDS_, and *l*_3′-UTR_ correspond to the length (nt) of the 5′- UTR, the coding sequence (CDS), and the 3′-UTR, respectively. To account for the potential underestimation of gene expression for samples with little or no 3′-UTR usage, we included the sample-specific PDUI values in the denominator as shown in the formula below:


$$\mathrm{PDUI}\;\mathrm{adjusted}\;\mathrm{counts}\;\mathrm{per}\;\mathrm{nt}=\frac{\mathrm{read}\;\mathrm{counts}}{l_{5^{\prime }-\mathrm{UTR}}+\;l_{\mathrm{CDS}}+(l_{3^{\prime }\text{-}\mathrm{UTR}}\times \mathrm{PDUI})}$$

Notice that if the entire 3′-UTR was fully used, (PDUI value equal to 1), the ‘PDUI adjusted counts per nt’ was identical to the ‘counts per nt’ measure. For each sample, we calculated the percentage expressed from *CALM1*, *CALM2*, and *CALM3* (with and without PDUI adjustment) since we wanted to determine the percentage expressed for each of the calmodulin coding genes rather than an absolute value of expression. The percentage of counts per calmodulin coding gene per sample was calculated as shown below:


$$\mathrm{Percentage}\;\mathrm{expressed}\;\mathrm{per}\;\mathrm{gene}=\;\frac{\mathrm{count}s_{i}}{\mathrm{count}s_{\mathrm{CALM}1}+\mathrm{count}s_{\mathrm{CALM}2}+\mathrm{count}s_{\mathrm{CALM}3}}\times 100$$

where counts*_i_*, corresponds to the counts of either *CALM1*, *CALM2*, or *CALM3*.

To validate our approach for PDUI adjustment, we compared the relative gene expression of *CALM1*, *CALM2*, and *CALM3* based on long-read and short-read sequencing data in paired samples from the GTEx project as described in Supplementary Methods.

As the percentages expressed per gene are statistically dependent as they sum to 100%, we applied compositional data analysis (reviewed in ref.^28^) The data was transformed using the isometric log-ratio transformation^29^ implemented in the compositions (version 2.0-9)^30^ package followed by the Hotelling’s T^2^ multivariate hypothesis test using the ICSNP package (version 1.1-2).^31^ Post hoc significance testing was conducted with one-sample t-tests using the *t.test()* function.

### Translation into calmodulin

The RNA-sequencing and ribosome profiling data was normalized together to enable integration of the two types of data for estimation of translational efficiency. The size factor was estimated to account for sequencing depths using the *estimateSizeFactorsForMatrix()* function from the DESeq2 R package (v.1.44.0).^32^ The reads were originally mapped to only the CDS of the genes.^18^ Since the CDS lengths of *CALM1*, *CALM2*, and *CALM3* are identical, we did not adjust the RNA-sequencing and ribosome profiling data for gene length. The translational efficiency was estimated for each gene per sample by taking the ratio of ribosome profiling counts over RNA sequencing counts as previously described.^18,33^

To obtain an estimate of the percentage of calmodulin that is translated from each of *CALM1*, *CALM2*, and *CALM3* in left ventricle tissue, we multiplied the ‘PDUI adjusted counts per nt’ obtained from GTEx v8 with the median translational efficiency for each of the genes. The compositional data analysis was conducted as described in the ‘Gene expression’ section.

### Ethics approval and consent to participate

The study was approved by the Institutional Review Board of Aalborg University (AAU031-1058687). The analyses were conducted on data that were either publicly available or available upon application (dbGaP: phs000424.v8.p2). The International Calmodulinopathy Registry study was approved by the Ethics Committees of the co-ordinating and enrolled centres.^7^ No additional data were generated for this study. The study was conducted in accordance with the Declaration of Helsinki.

## Results

### Expressed transcripts of *CALM1*, *CALM2*, and *CALM3*

**Figure 2:**
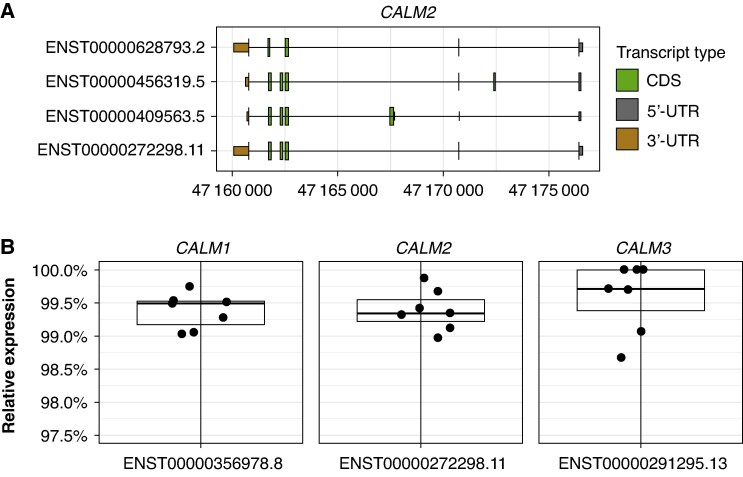
Left ventricle transcript expression. (*A*) Coding sequence (CDS) and untranslated regions (UTR) of the protein-coding transcripts of the *CALM2* gene. (*B*) Relative expression of the most expressed transcript for each of *CALM1*, *CALM2*, and *CALM3* in seven left ventricle samples.

We used the GENCODE v26 annotation file to compare the genetic structure of the *CALM1*, *CALM2*, and *CALM3* genes. There were 6, 4, and 7 different protein-coding transcripts for *CALM1*, *CALM2*, and *CALM3*, respectively (*Figure 2A* and Supplementary material online, Figure  *S1*).

To quantify transcript expression, we used the long-read RNA sequencing data of 66 samples that represented 13 different tissues and 46 donors from GTEx (v9).^16^ Among the seven left ventricle samples, a single dominant transcript per gene (*CALM1*: ENST00000356978.8, *CALM2*: ENST00000272298.11, and *CALM3*: ENST00000291295.13) accounted for 98.7–100% of all transcripts (*Figure 2B*). These transcripts corresponded to the Matched Annotation from NCBI and EMBL-EBI (MANE)^21^ transcripts of *CALM1*, *CALM2*, and *CALM3*. Among all samples, the median percentage expressed by the MANE transcripts were 99.3% for *CALM1* (range: 96.9–100%), 99.5% for *CALM2* (range: 98.3–100%), and 99.3% for *CALM3* (range: 98.3–100%) (see Supplementary material online, *Table S1*). Due to the low expression level of the remaining transcripts, we excluded these from the rest of the analyses.

### Different usage of the 3′-untranslated regions and gene expression among *CALM1*, *CALM2*, and *CALM3*

**Figure 3:**
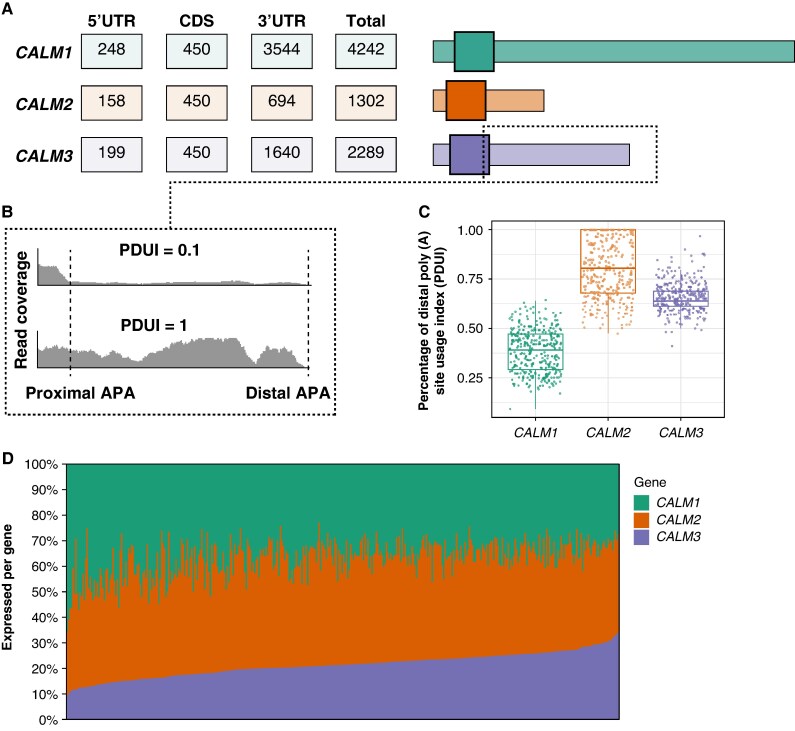
*CALM1*, *CALM2*, and *CALM3* transcript structures and alternative polyadenylation usage. (*A*) The lengths (in nucleotides) of the 5′-UTRs, the coding sequence (CDS) and the 3′-UTR among *CALM1*, *CALM2*, and *CALM3* together with an illustration of the transcript lengths. (*B*) Example of read coverage across the 3′-UTR for *CALM3* with proximal and distal alternative polyadenylation (APA) sites indicated by dashed lines. Percentage of distal polyA site usage index (PDUI) values represents the relative usage of distal polyadenylation sites, where higher values indicate greater distal site preference. (*C*) *CALM1*, *CALM2*, and *CALM3* PDUI values obtained from left ventricle tissue. (*D*) Gene expression per individual in left ventricle tissue where each bar represents the percentage expressed compared with the total *CALM1*, *CALM2*, and *CALM3* expression. The gene expression measures were adjusted for 3′-UTR usage.

The total length of the transcripts (including 5′-UTR, CDS, and 3′-UTR) ranged from 1302 to 4242 nucleotides (nt) among the MANE transcripts of *CALM1*, *CALM2*, and *CALM3* (*Figure 3A*). The CDS lengths were identical for all three genes (450 nt including start and stop codons), while the length of the 3′-UTRs was 3544 nt for *CALM1*, 694 nt for *CALM2*, and 1640 nt for *CALM3*.

Commonly used measures for comparison of RNA sequencing data account for total gene length as a part of the normalization procedure.^34^ However, the full-length 3′-UTRs are not necessarily expressed. Consequently, gene expression measures may be inaccurate for genes with relative long 3′UTRs if the 3′-UTR usage is not taken into account. To address this challenge, we evaluated the 3′-UTR usage by calculating the PDUI values for *CALM1*, *CALM2*, and *CALM3* (*Figure 3B* and *C* and Supplementary material online, Figure  *S2*). The analysis was conducted on 15 201 samples from 838 individuals for a total of 49 different tissues using the short-read RNA sequencing data of GTEx (v8) as previously described.^25^ As shown in *Figure 3C*, the PDUI values ranged from 0.09 to 0.64 for *CALM1* (median: 0.39), from 0.48 to 1 for *CALM2* (median PDUI: 0.81), and from 0.41 to 0.97 for *CALM3* (median PDUI: 0.64) in left ventricle tissue. Among all tissues, PDUI values ranged from 0.03 to 1 for *CALM1* (median: 0.32), from 0.235 to 1 for *CALM2* (median: 0.70), and from 0.12 to 1 for *CALM3* (median: 0.69) (see Supplementary material online, Figure  *S2*).

Based on the PDUI-adjusted gene expression values, we calculated the relative gene expression of *CALM1*, *CALM2*, and *CALM3* among each tissue (see Supplementary material online, Figure  *S3*). In left ventricle tissue, the overall gene expression composition of *CALM1*, *CALM2*, and *CALM3* differed from a uniform distribution (Hotelling’s T^2^, *P* < 2 × 10).^16^ Post hoc analyses revealed that the mean expression percentage of *CALM2* (41.9%) was significantly higher compared with *CALM1* (36.8%) (*P* < 2 × 10^−16^) and that *CALM3* was significantly less expressed compared with *CALM1* and *CALM2* (*P* < 2 × 10^−16^) (*Figure 3D*). Furthermore, *CALM2* was the most expressed gene in 47 out of 49 tissues (see Supplementary material online, Figure  *S3*). *CALM1* was the least expressed gene in 10 out of 49 tissues while *CALM3* was the least expressed in the remaining 39 tissues.

### Translational efficiency of *CALM1*, *CALM2*, and *CALM3* in the left ventricle

**Figure 4:**
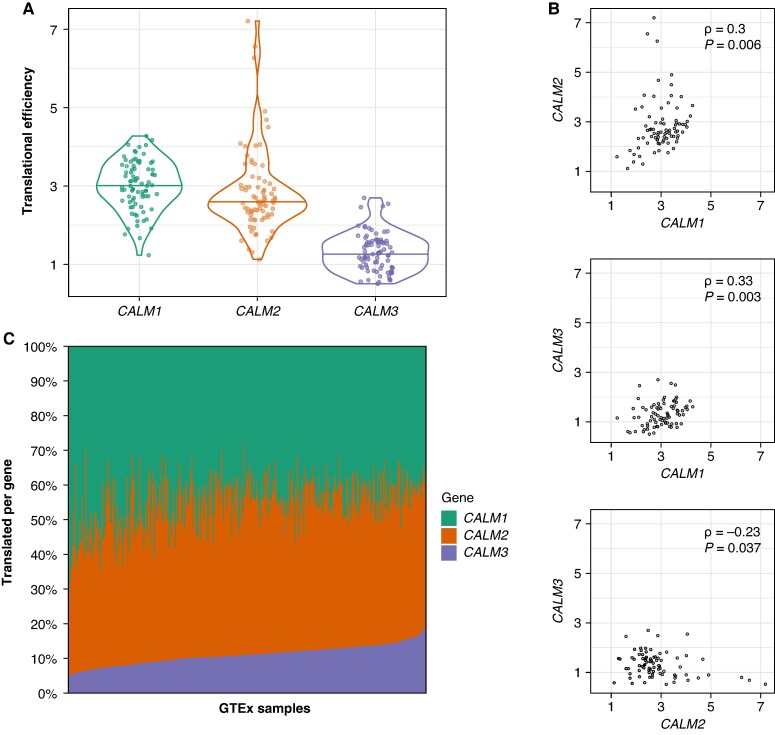
Estimation of translation among *CALM1*, *CALM2*, and *CALM3* in left ventricle. (*A*) Violin plots of translational efficiency per individual among *CALM1*, *CALM2*, and *CALM3* in 80 left ventricle samples. The translational efficiency was significantly different using the Wilcoxon signed rank test for *CALM1* vs. *CALM2* (*P* = 2.53 × 10^−3^), *CALM1* vs. *CALM3* (*P* = 9.31 × 10^−15^), and for *CALM2* vs. *CALM3* (*P* = 2.76 × 10^−14^). (*B*) Pairwise comparisons of translational efficiency. (*C*) Estimated percentage of calmodulin translated per gene for left ventricle samples obtained from Genotype-Tissue Expression (GTEx) project.

To further examine to which extent the *CALM1*, *CALM2*, and *CALM3* genes contribute to the total calmodulin amount, we calculated the translational efficiency i.e. the number of ribosomes per transcript, based on a previous investigation using paired RNA sequencing and ribosome profiling data from left ventricle tissue.^18^ The median translational efficiency varied among the three genes, corresponding to 3.01 for *CALM1*, 2.59 for *CALM2*, and 1.26 for *CALM3* (*Figure 4A*). Compared with *CALM3,* the translational efficiency of *CALM1* and *CALM2* was 137.5% and 101.6% higher, respectively. A similar tendency was observed for translational efficiency stratified by sex and presence or absence of dilated cardiomyopathy (see Supplementary material online, Figure  *S4*). *CALM3* was consistently expressed at significantly lower levels than *CALM1* and *CALM2*, and the order of the translational efficiency (*CALM1* > *CALM2* > *CALM3*) was preserved.

Next, we conducted three pairwise comparisons of the correlations of translational efficiency among all individuals. We observed a significant positive correlation after correction for multiple testing between *CALM1* and *CALM2* (Spearman’s *ρ* = 0.3, *P* < 0.017), and between *CALM1* and *CALM3* (Spearman’s *ρ* = 0.33, *P* < 0.017) but not between *CALM2* and *CALM3* (Spearman’s *ρ* = −0.23, *P* = 0.037) (*Figure 4B*).

Last, we integrated gene expression data with median translational efficiencies to estimate the percentage of calmodulin translated per left ventricle sample from each of *CALM1*, *CALM2*, and *CALM3* (*Figure 4C*). The overall composition of calmodulin translated from *CALM1*, *CALM2*, and *CALM3* differed from a uniform distribution (Hotelling’s T^2^, *P* < 2 × 10^−16^). Post hoc analyses indicated no difference in the percentage of calmodulin translated from *CALM1* (mean: 44.8%, range: 28.4–71.9%) and *CALM2* (mean: 44.2% range: 23.9%–63.6%) (*P* = 0.44). The percentage of calmodulin translated from *CALM3* (mean: 11.0%, range 4.2–18.9%) was significantly lower compared with *CALM1* and *CALM2* (*P* < 2 × 10^−16^).

Assuming equal expression and translation of both alleles for each of the *CALM1*, *CALM2*, or *CALM3* genes, the percentage of calmodulin protein carrying a missense mutation is estimated to be, on average, 23%, 22%, and 6% of the total calmodulin amount.

### Differential consequence of DNA variants in *CALM1*, *CALM2*, and *CALM3*

**Figure 5:**
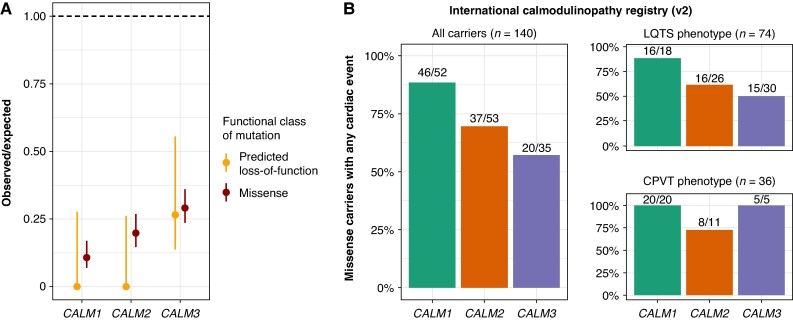
Consequence of DNA variants in *CALM1*, *CALM2*, and *CALM3*. (*A*) Observed-to-expected ratios and 90% confidence intervals of *CALM1*, *CALM2*, and *CALM3* variants in gnomAD categorized by functional class. The horizontal dashed line at observed-to-expected = 1 represents the expected number of variants under neutral selection. (*B*) Percentage of missense variant carriers in *CALM1*, *CALM2*, and *CALM3* who exhibit symptoms, based on data from the International Calmodulinopathy Registry. Any cardiac event corresponds to any of arrhythmic syncope, aborted cardiac arrest, sudden cardiac death, or appropriate implantable cardioverter–defibrillator shocks. The distributions are shown for all carriers (left panel) and the two subgroups, long QT syndrome (LQTS) and catecholaminergic polymorphic ventricular tachycardia (CPVT) (right panels).

To assess the selection against DNA variants, we compared the observed-to-expected ratios of variants in gnomAD. An observed-to-expected ratio equal to 1 indicates that there is no selection against that particular type of variant while an observed-to-expected ratio close to zero indicates strong negative selection meaning that harmful variants are removed from the population because they reduce survival or reproductive success. The observed-to-expected ratios of missense variants were 0.11 (90% CI, 0.07–0.17), 0.20 (90% CI, 0.15–0.27), and 0.29 (90% CI, 0.23–0.36) for *CALM1*, *CALM2*, and *CALM3*, respectively (*Figure 5A*). Furthermore, the observed-to-expected ratio of predicted loss-of-function mutations was 0.27 (90% CI, 0.14–0.56) for *CALM3* while it was 0 (no observations) for both *CALM1* (90% CI, 0–0.28) and *CALM2* (90% CI, 0–0.26) (*Figure 5A*).

To investigate the clinical characteristics of DNA variants in *CALM1*, *CALM2*, and *CALM3*, we used the data from 140 individuals with a missense variant in the International Calmodulinopathy Registry.^7^ There was a significant association between the calmodulin-encoding genes and the fraction of individuals with any cardiac event (Chi-squared test, *P* = 0.004). As shown in *Figure 5B*, the percentage of carriers with a cardiac event was highest for *CALM1* (46/52, 89%) followed by *CALM2* (37/53, 70%) and *CALM3* (20/35, 57%). We further investigated the events among two major subgroups of missense carriers with either LQTS (*n* = 74) or CPVT (*n* = 36). The test results were not significant for the LQTS (Fisher’s Exact test, *P* = 0.02) nor the CPVT subgroups (Fisher’s Exact test, *P* = 0.04) when using the multiple testing corrected threshold of (*P* = 0.05/3–0.017). However, we observed that the variant distribution in the LQTS subgroup reflected the distribution among all patients in contrast to the CPVT subgroup. Predicted loss-of-function variants (incl. stop, frameshift or splice site variants) were not observed in any of *CALM1*, *CALM2*, and *CALM3* in the International Calmodulinopathy Registry.

## Discussion

Despite encoding identical amino acid sequences, the negative selection and clinical characteristics vary greatly for *CALM1*, *CALM2*, and *CALM3*. In this study, we identified key differences in 3′ UTR usage, gene expression, and translational efficiency based on our multi-omics approach for investigating the role of *CALM1*, *CALM2*, and *CALM3*.

### Gene-specific consequences of missense mutations

Risk stratification of calmodulinopathy patients often relies on the comparison of the same missense variants in one of the other two calmodulin-encoding genes due to the extreme rarity of the missense variants. We show that *CALM3* is generally subject to less negative selection than *CALM1* and *CALM2* (*Figure 5A*) and that the cardiac events are more prevalent among *CALM2* and, especially, *CALM1* carriers (*Figure 5B*). This trend was predominant among individuals with the LQTS phenotype suggesting that the molecular disease mechanism that drives the CPVT phenotype in calmodulinopathy is different and less sensitive towards the fraction of mutated calmodulin to normal calmodulin, compared with the LQTS disease mechanism.

### Expression and translational efficiency are coupled to gene-specific consequence of missense mutations

We obtained considerable insight into the roles *CALM1*, *CALM2*, and *CALM3* by the investigation of the RNA sequencing and ribosome profiling data. First, the MANE transcripts constitute ∼99% of all transcripts among each of *CALM1*, *CALM2*, and *CALM3* (*Figure 2B* and Supplementary material online, *Table S1*). Due to the low expression in the remaining transcripts, variants in unique exons of these transcripts (e.g. ENST00000409563.5 of *CALM2*, *Figure 2A*) are unlikely to exert any phenotypic effect based on alterations in the calmodulin protein and should therefore be interpreted with extreme caution in a clinical context.

Second, *CALM3* was previously shown to be the most expressed calmodulin-encoding gene in the left ventricle.^35^ In contrast, we show that *CALM3* is the least expressed calmodulin-encoding gene in left ventricle tissue (*Figure 3D*). We consider that different primer efficiencies for the quantitative reverse transcription polymerase chain reaction of *CALM1*, *CALM2*, and *CALM3* in the original study may be a possible cause of this discordance.

Third, we provide the first evidence that *CALM1* and *CALM2* contribute substantially more than *CALM3* to the translation into calmodulin in left ventricle tissue (*Figure 4C*) in line with the observation that *CALM3* missense mutations are under less negative selection (*Figure 5A*) and that cardiac events are less prevalent among *CALM3* missense carriers (*Figure 5B*). Since the dosage of mutated calmodulin is linked to the severity of arrhythmias in mice,^12^ we consider the possibility that reduced dosage of mutated calmodulin from *CALM3* might contribute to a less severe phenotype for *CALM3* missense mutation carriers. Our coupled usage of clinical, genetic, and transcriptomic data provides a unique opportunity, even in an ultra-rare disease, to unravel the genotype-phenotype correlation.

### Variable expressivity is possibly caused by variable contribution to calmodulin

Based on the integration of RNA sequencing and ribosome profiling data, we provide novel and unique evidence for the variable contribution from each of *CALM1*, *CALM2*, and *CALM3* to the total protein amount. For instance, the estimated *CALM3* contribution to the total calmodulin amount ranged from 4% to 19% corresponding to more than a four-fold difference among the individuals. This observation is crucial considered that the clinical manifestation of calmodulinopathy may be asymptomatic or severe in the form of sudden cardiac death even within the same family.^8^ We reason that the variable dosage of mutated calmodulin compared with normal calmodulin is partly responsible for the variable expressivity of symptoms within families affected by calmodulinopathy.

### Usage of 3′-untranslated regions in the heart

We profiled the 3′-UTR usage among 49 human tissues from GTEx. Consistent with previous investigations of the 3′-UTR of *CALM1* in rodents,^36,37^ we observed increased usage of the long 3′-UTR in brain tissues compared with other tissues (see Supplementary material online, Figure  *S2*). Our findings demonstrate important technical implications of variable 3′-UTR usage when comparing gene expression. The quantification bias was particularly pronounced for *CALM1* due to the variable usage and the greater length of the 3′-UTR (see Supplementary material online, Figure  *S3*). Furthermore, we also show that the usage of the 3′-UTR is different among *CALM1*, *CALM2*, and *CALM3* in left ventricle tissue (*Figure 3C*). It was recently shown that murine *Calm1*, *Calm2*, and *Calm3* are localized different within cardiomyocytes.^38^ Since the 3′-UTRs of calmodulin-encoding genes are linked to different intracellular localisations,^36,37^ our findings of variable 3′-UTR usage within and among *CALM1*, *CALM2*, and *CALM3* may be linked to spatial distribution in cardiomyocytes.

### Co-regulation of translational efficiency

Novel therapeutic approaches for calmodulinopathy rely on the depletion of the gene affected by a missense variant.^10,11,39^ Specifically, Bortolin *et al*.^10^ showed that the total calmodulin levels are maintained after *Calm1* depletion due to compensatory regulations of *Calm2* and *Calm3* transcript levels. We examined the pairwise correlations of translational efficiencies among *CALM1*, *CALM2*, and *CALM3* to test if relatively low translational efficiency in one gene was compensated by an increase in the other two genes, as a mechanism for ensuring a particular level of calmodulin protein. In such cases, a negative correlation between translational efficiencies would have been observed. However, we only observed a relatively modest negative correlation between the translational efficiencies of *CALM2* and *CALM3*, and even positive correlations for *CALM1* vs. *CALM2* and for *CALM1* vs. *CALM3* (*Figure 4B*). This suggests limited or no translational compensation, but rather co-regulation at the translational level for *CALM1* and the other two *CALM* genes.

### Limitations

In this study, we focused on the cardiac features of calmodulinopathy but acknowledge that the neurodevelopmental features may also be the cause of the observed negative selection of *CALM1*, *CALM2*, and *CALM3* variants. Since individual-level phenotypic characteristics were unavailable from the gnomAD cohort, we were unable to investigate if the selection was driven by neurodevelopmental, cardiac or yet undescribed symptoms. Furthermore, as patients in the International Calmodulinopathy Registry are primarily presenting with arrhythmic rather than neurodevelopmental symptoms, we were unable to estimate if neurodevelopmental symptoms were overrepresented in any of the genes in this cohort. Our study was based on bulk RNA sequencing and ribosome profiling data from individuals without calmodulin missense mutation. While we gained considerable insight into the general role of *CALM1*, *CALM2*, and *CALM3*, we argue that future studies should be designed to address the diversity of expression among single cells in individuals with and without calmodulin missense mutations.

## Conclusion

Overall, our work suggests that *CALM1*, *CALM2*, and *CALM3* exert different roles. Although missense variants in *CALM3* are generally pathogenic, we provide the first evidence that the consequence of missense variants in *CALM3* is less severe compared with missense variants in *CALM1* and *CALM2*, primarily due to fewer cardiac events among individuals with the LQTS phenotype of calmodulinopathy.

## Supplementary Material

euag052_Supplementary_Data

## Data Availability

The constraint metrics data file (gnomad.v4.1.constraint_metrics.tsv) are available from https://gnomad.broadinstitute.org. Data from the International Calmodulinopathy Registry are available from the online version of the paper published in the European Heart Journal (https://doi.org/10.1093/eurheartj/ehad418). The mapped read counts from GTEx were downloaded from the GTEx Portal. Counts from the short-read sequencing data (v8 release) was downloaded from: https://gtexportal.org/home/downloads/adult-gtex/bulk_tissue_expression. The long-read RNA sequencing data (v9 release) was downloaded from https://gtexportal.org/home/downloads/adult-gtex/long_read_data. The raw reads for the calculation of the PDUI values were obtained from dbGaP (accession number phs000424.v8.p2). The GENCODE (v26) annotation file was downloaded from: https://ftp.ebi.ac.uk/pub/databases/gencode/Gencode_human/release_26/. The RNA-sequencing and ribosome profiling data from the 80 left ventricle samples is available from a web application (https://shiny.mdc-berlin.de/cardiac-translatome/) and in the online version of the paper published in Cell (https://doi.org/10.1016/j.cell.2019.05.010). The code for the analyses and the figures are available from GitHub (https://github.com/SteffanChristiansen/calmodulinopathy_var_exp_te) and Zenodo (https://zenodo.org/records/15574676).

## References

[1] Schwartz PJ, Crotti L. Calmodulinopathies: the need for a registry. Circ: Genom Precis Med 2025;18:e005503. 41191520 10.1161/CIRCGEN.125.005503 PMC12711277

[2] Schwartz PJ, Crotti L. Calmodulinopathies: the need for a registry. Europace 2025;27:euaf158. 41206343 10.1093/europace/euaf158 PMC12596160

[3] Schwartz PJ, Crotti L. Calmodulinopathies the need for a registry. JACC Clin Electrophysiol 2025;27:euaf158. 10.1016/j.jacep.2025.08.004 40900065

[4] Schwartz PJ, Crotti L. Long QT syndrome. N Engl J Med 2025;393:2023–34. 41259757 10.1056/NEJMra2400853

[5] Nyegaard M, Overgaard MT, Søndergaard MT, Vranas M, Behr ER, Hildebrandt LL et al Mutations in calmodulin cause ventricular tachycardia and sudden cardiac death. Am J Hum Genet 2012;91:703–12. 23040497 10.1016/j.ajhg.2012.08.015 PMC3484646

[6] Crotti L, Spazzolini C, Tester DJ, Ghidoni A, Baruteau A-E, Beckmann B-M et al Calmodulin mutations and life-threatening cardiac arrhythmias: insights from the International Calmodulinopathy Registry. Eur Hear J 2019;40:2964–75. 10.1093/eurheartj/ehz311 PMC6748747 31170290

[7] Crotti L, Spazzolini C, Nyegaard M, Overgaard MT, Kotta M, Dagradi F et al Clinical presentation of calmodulin mutations: the International Calmodulinopathy Registry. Eur Heart J 2023;44:3357–70. 37528649 10.1093/eurheartj/ehad418 PMC10499544

[8] Kato K, Isbell HM, Fressart V, Denjoy I, Debbiche A, Itoh H et al Novel CALM3 variant causing calmodulinopathy with Variable expressivity in a 4-generation family. Circ Arrhythm Electrophysiol 2022;15:E010572. 35225649 10.1161/CIRCEP.121.010572

[9] Friedberg F, Rhoads AR. Evolutionary aspects of calmodulin. IUBMB Life 2001;51:215–21. 11569915 10.1080/152165401753311753

[10] Bortolin RH, Nawar F, Park C, Trembley MA, Prondzynski M, Sweat ME et al Antisense oligonucleotide therapy for calmodulinopathy. Circulation 2024;150:1199–210. 39155863 10.1161/CIRCULATIONAHA.123.068111 PMC11747850

[11] Limpitikul WB, Dick IE, Tester DJ, Boczek NJ, Limphong P, Yang W et al A precision medicine approach to the rescue of function on malignant calmodulinopathic long-QT syndrome. Circ Res 2017;120:39–48. 27765793 10.1161/CIRCRESAHA.116.309283 PMC5516949

[12] Tsai W-C, Yang C-F, Lin S-Y, Liang S-Y, Tsai W-C, Guo S et al Cardiac enrichment of mutant calmodulin protein in a murine model of a human calmodulinopathy. JCI Insight 2025;10. https://insight.jci.org/articles/view/185524#FN Version 2 (September 9, 2025): Electronic publication 10.1172/jci.insight.185524 PMC12487685 40705464

[13] Lonsdale J, Thomas J, Salvatore M, Phillips R, Lo E, Shad S et al The Genotype-Tissue Expression (GTEx) project. Nat Genet 2013;45:580–5. 23715323 10.1038/ng.2653 PMC4010069

[14] Chen S, Francioli LC, Goodrich JK, Collins RL, Kanai M, Wang Q et al A genomic mutational constraint map using variation in 76,156 human genomes. Nature 2024;625:92–100. 38057664 10.1038/s41586-023-06045-0 PMC11629659

[15] Carithers LJ, Ardlie K, Barcus M, Branton PA, Britton A, Buia SA et al A novel approach to high-quality postmortem tissue procurement: the GTEx project. Biopreserv Biobank 2015;13:311–9. 26484571 10.1089/bio.2015.0032 PMC4675181

[16] Glinos DA, Garborcauskas G, Hoffman P, Ehsan N, Jiang L, Gokden A et al Transcriptome variation in human tissues revealed by long-read sequencing. Nature 2022;608:353–9. 35922509 10.1038/s41586-022-05035-y PMC10337767

[17] Consortium G . The GTEx consortium atlas of genetic regulatory effects across human tissues. Science 2020;369:1318–30. 32913098 10.1126/science.aaz1776 PMC7737656

[18] van Heesch S, Witte F, Schneider-Lunitz V, Schulz JF, Adami E, Faber AB et al The translational landscape of the human heart. Cell 2019;178:242–260.e29. 31155234 10.1016/j.cell.2019.05.010

[19] Ingolia NT, Ghaemmaghami S, Newman JRS, Weissman JS. Genome-wide analysis in vivo of translation with nucleotide resolution using ribosome profiling. Science 2009;324:218–23. 19213877 10.1126/science.1168978 PMC2746483

[20] Karczewski KJ, Francioli LC, Tiao G, Cummings BB, Alföldi J, Wang Q et al The mutational constraint spectrum quantified from variation in 141,456 humans. Nature 2020;581:434–43. 32461654 10.1038/s41586-020-2308-7 PMC7334197

[21] Morales J, Pujar S, Loveland JE, Astashyn A, Bennett R, Berry A et al A joint NCBI and EMBL-EBI transcript set for clinical genomics and research. Nature 2022;604:310–5. 35388217 10.1038/s41586-022-04558-8 PMC9007741

[22] Wickham H, Averick M, Bryan J, Chang W, McGowan L, François R et al Welcome to the tidyverse. J Open Source Softw 2019;4:1686.

[23] Pedersen TL. patchwork: The Composer of Plots. 2024. https://github.com/thomasp85/patchwork

[24] Gustavsson EK, Zhang D, Reynolds RH, Garcia-Ruiz S, Ryten M. Ggtranscript: an R package for the visualization and interpretation of transcript isoforms using ggplot2. Bioinformatics 2022;38:3844–6. 35751589 10.1093/bioinformatics/btac409 PMC9344834

[25] Cui Y, Peng F, Wang D, Li Y, Li JS, Li L et al 3′aQTL-atlas: an atlas of 3′UTR alternative polyadenylation quantitative trait loci across human normal tissues. Nucleic Acids Res 2021;50:D39–45. 10.1093/nar/gkab740 PMC8728222 34432052

[26] Li L, Huang K-L, Gao Y, Cui Y, Wang G, Elrod ND et al An atlas of alternative polyadenylation quantitative trait loci contributing to complex trait and disease heritability. Nat Genet 2021;53:994–1005. 33986536 10.1038/s41588-021-00864-5

[27] Feng X, Li L, Wagner EJ, Li W. TC3A: the cancer 3′ UTR atlas. Nucleic Acids Res 2018;46:D1027–30. 30053266 10.1093/nar/gkx892 PMC5753254

[28] Greenacre M . Compositional data analysis. Annu Rev Stat Appl 2021;8:271–99.

[29] Egozcue JJ, Pawlowsky-Glahn V, Mateu-Figueras G, Barceló-Vidal C. Isometric logratio transformations for compositional data analysis. Math Geol 2003;35:279–300.

[30] van den Boogaart KG, Tolosana-Delgado R, Bren M. compositions: Compositional Data Analysis. 2025. https://cran.r-project.org/web/packages/compositions/index.html

[31] Nordhausen K, Sirkia S, Oja H, Tyler DE. ICSNP: Tools for Multivariate Nonparametrics. 2023. https://cran.r-project.org/web/packages/ICSNP/index.html

[32] Love MI, Huber W, Anders S. Moderated estimation of fold change and dispersion for RNA-Seq data with DESeq2. Genome Biol 2014;15:550. 25516281 10.1186/s13059-014-0550-8 PMC4302049

[33] Chothani S, Adami E, Ouyang JF, Viswanathan S, Hubner N, Cook SA et al deltaTE: detection of translationally regulated genes by integrative analysis of Ribo-seq and RNA-seq data. Curr Protoc Mol Biol 2019;129:e108. 31763789 10.1002/cpmb.108 PMC9285699

[34] Zhao Y, Li M-C, Konaté MM, Chen L, Das B, Karlovich C et al TPM, FPKM, or normalized counts? A comparative study of quantification measures for the analysis of RNA-seq data from the NCI Patient-Derived Models Repository. J Transl Med 2021;19:269. 34158060 10.1186/s12967-021-02936-w PMC8220791

[35] Crotti L, Johnson CN, Graf E, Ferrari GM, Cuneo BF, Ovadia M et al Calmodulin mutations associated with recurrent cardiac arrest in infants. Circulation 2013;127:1009–17. 23388215 10.1161/CIRCULATIONAHA.112.001216 PMC3834768

[36] Bongmin BAE, Gruner HN, Lynch M, Feng T, Kevin SO, Oliver D et al Elimination of Calm1 long 3′-UTR mRNA isoform by CRISPR-Cas9 gene editing impairs dorsal root ganglion development and hippocampal neuron activation in mice. RNA 2020;26:1414–30. 32522888 10.1261/rna.076430.120 PMC7491327

[37] Tushev G, Glock C, Heumüller M, Biever A, Jovanovic M, Schuman EM. Alternative 3′ UTRs modify the localization, regulatory potential, stability, and plasticity of mRNAs in neuronal compartments. Neuron 2018;98:495–511.e6. 29656876 10.1016/j.neuron.2018.03.030

[38] Bogdanov V, Mariangelo JIE, Soltisz AM, Sakuta G, Pokrass A, Beard C et al Distinct intracellular spatiotemporal expression of Calmodulin genes underlies functional diversity of CaM-dependent signaling in cardiac myocytes. Cardiovasc Res 2025;121:1052–65. 40273382 10.1093/cvr/cvaf059 PMC12641389

[39] Hamrick SK, Kim CSJ, Tester DJ, Gencarelli M, Tobert KE, Gluscevic M et al Single construct suppression and replacement gene therapy for the treatment of all CALM1 -, CALM2 -, and CALM3 -mediated arrhythmia disorders. Circ: Arrhythmia Electrophysiol 2024:e012036. 10.1161/CIRCEP.123.012036 39069900

