# Supplementary material for: CALM1, CALM2, and CALM3 expression and translation efficiency provide insight into the severity of calmodulinopathy
Source: Europace. 2026 Mar 18;28(4):euag052. doi: 10.1093/europace/euag052 (PMC13127890; doi:10.1093/europace/euag052)
Supplement: euag052_Supplementary_Data [file euag052_supplementary_data.zip › Supplementary Material - 2026.02.19.docx]

Supplementary Material

[Supplementary methods 2](#_Toc221689158)

[Adjustment for percentage of distal poly(A) site usage index 2](#_Toc221689159)

[Supplementary figures 4](#_Toc221689160)

[Supplementary figure 1 4](#_Toc221689161)

[Supplementary figure 2 5](#_Toc221689162)

[Supplementary figure 3 6](#_Toc221689163)

[Supplementary figure 4 8](#_Toc221689164)

# Supplementary methods

## Adjustment for percentage of distal poly(A) site usage index

Due to the variable usage of the 3’-UTRs, we investigated two different approaches for comparison of gene expression ("counts per nt" and "PDUI adjusted counts per nt") among *CALM1*, *CALM2*, and *CALM3*. To illustrate the effect of accounting for PDUI values, we constructed an example of *CALM1* expression in two different artificial samples that differed solely in their PDUI values (Fig S3A). In sample #1, the full-length 3’-UTR was used (PDUI = 1), resulting in no discrepancy between the values reported as "counts per nt" and "PDUI adjusted counts per nt". In contrast, in sample #2, where the full-length 3-’UTR was not used (PDUI = 0), the "PDUI adjusted counts per nt" exhibited a 6.1-fold increase relative to the unadjusted "counts per nt" even though sample #2 represents the same number of RNA copies as sample #1.

We tested our approach by comparing the percentage of counts that could be attributed to each of *CALM1*, *CALM2*, and *CALM3* with and without PDUI adjustment for the subset of 61 GTEx samples with both short-read and long-read RNA sequencing data (Fig S3B). The shortest distance between each data point (x_1_, y_1_) and the identity line (y = x) was calculated as the absolute value of y_1_- x_1_ divided by the square root of (1 + 1).

The median distances to the identity line were significantly different (paired Wilcoxon rank sum test) for *CALM1* (*p* = 2.81×10^-7^), *CALM2* (*p* = 4.95×10^-7^), and *CALM3* (*p* = 3.77×10^-4^). The median distance was reduced with 5.8 percentage points for *CALM1* (27.6%), 3.3 percentage points for *CALM2* (29.8%), and 0.6 percentage points for *CALM3* (6.7%) by using “PDUI adjusted counts per nt” compared to the “counts per nt” (Fig S3B).

The code for the analyses and the figures are available from GitHub (<https://github.com/SteffanChristiansen/calmodulinopathy_var_exp_te>) and Zenodo (https://zenodo.org/records/15574676).

# Supplementary figures

## Supplementary figure 1

| 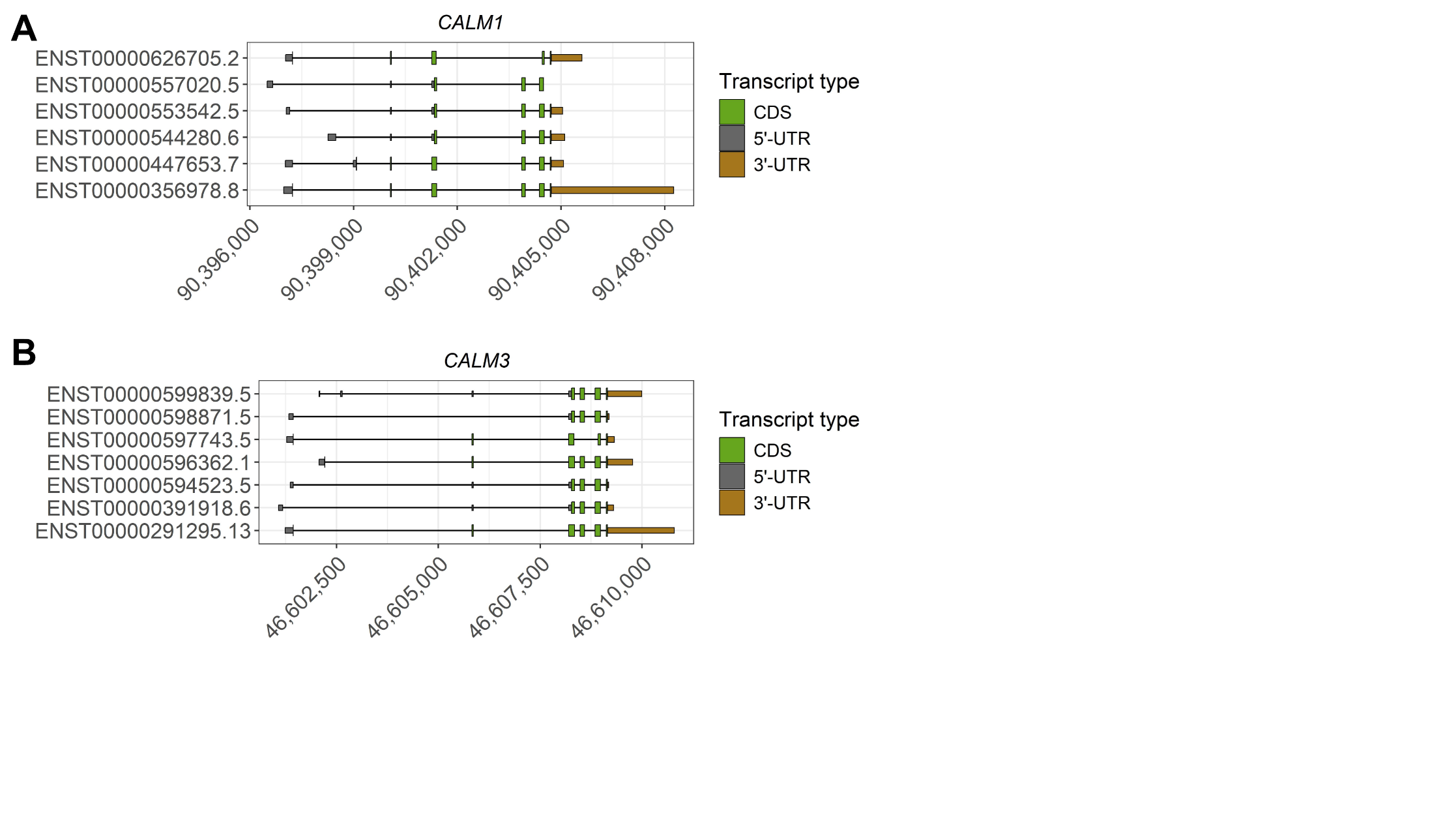 |
| --- |
| **Supplementary figure 1 \| Structure of *CALM1* and *CALM3*.** Coding sequence (CDS) and untranslated regions (UTR) of the protein-coding transcripts in A) *CALM1* and B) *CALM3*. |

## Supplementary figure 2

| 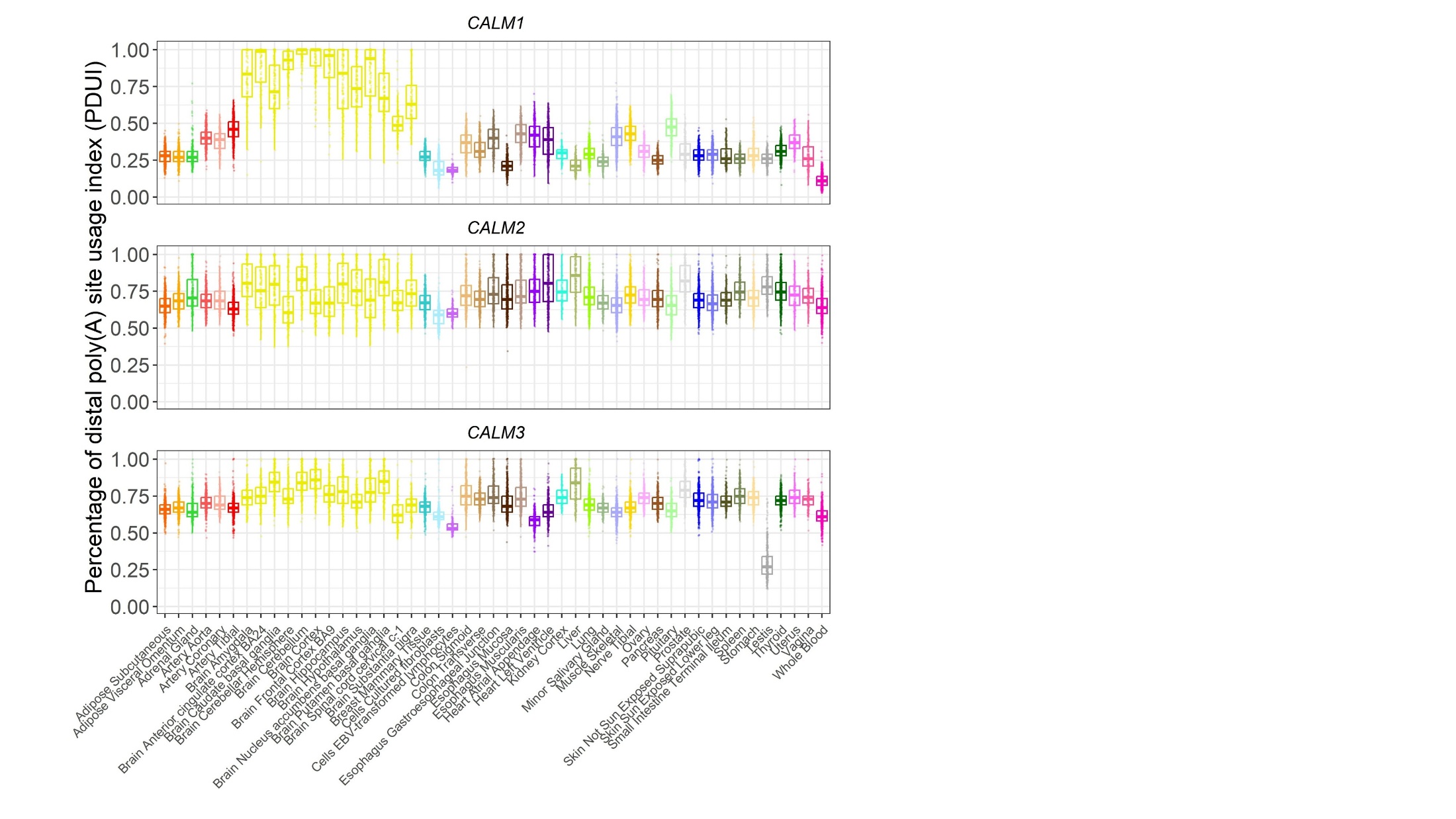 |
| --- |
| **Supplementary figure 2 \| Percentage of distal poly(A) usage index values among *CALM1***, ***CALM2*, and *CALM3*.**  Box plots of percentage of distal poly(A) usage index values (PDUI) per tissue among *CALM1*, *CALM2*, and *CALM3.* Each data point corresponds to a sample-specific and gene-specific PDUI value. The colour codes indicate tissue of origin and are adopted from the GTEx project. |

## Supplementary figure 3

| 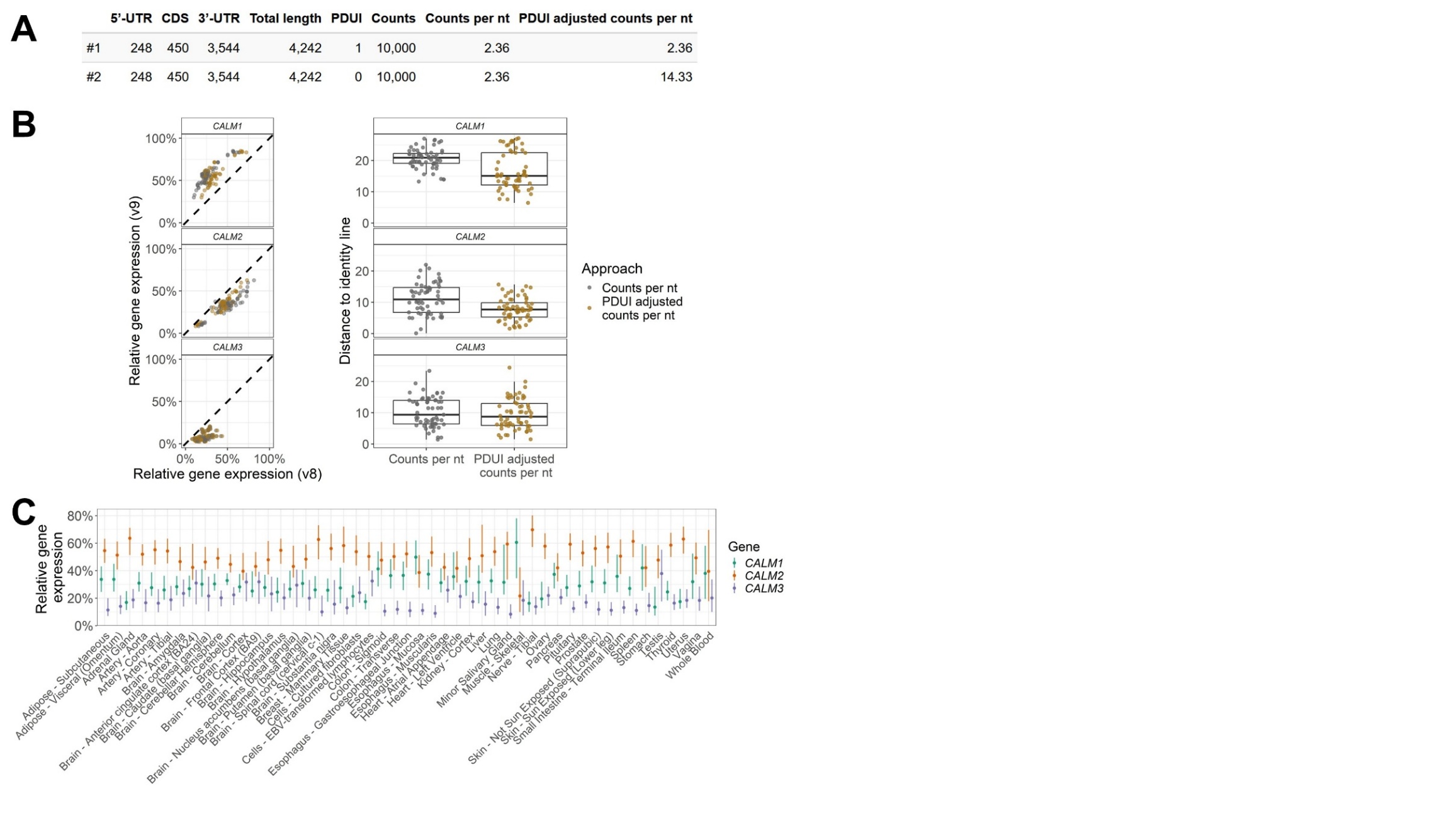 |
| --- |

**Supplementary figure 3 | Impact of 3’-untranslated region usage on gene expression values.**

A) Constructed example on the effect of 3’-untranslated region (UTR) usage estimated by percentage of distal poly(A) usage site index (PDUI) values. The lengths of the 5’-UTR, coding sequence (CDS), and 3’-UTR of *CALM1* was obtained from GENCODE v26. B) The left panels show the relative expression per sample for long-read sequencing data (v9) and short-read sequencing data (v8) in paired samples. Perfectly matching values are expected to overlap with the identify line (dashed). The right panels show the corresponding distances to the identity line for the data in the left panel. C) Percentage expressed of *CALM1*, *CALM2*, and *CALM3* per tissue among the 49 examined tissues based on PDUI adjusted counts per nt. The median values are visualised by dots and the whiskers represent the limits of the 2.5%-97.5% percentile range.

## Supplementary figure 4

| 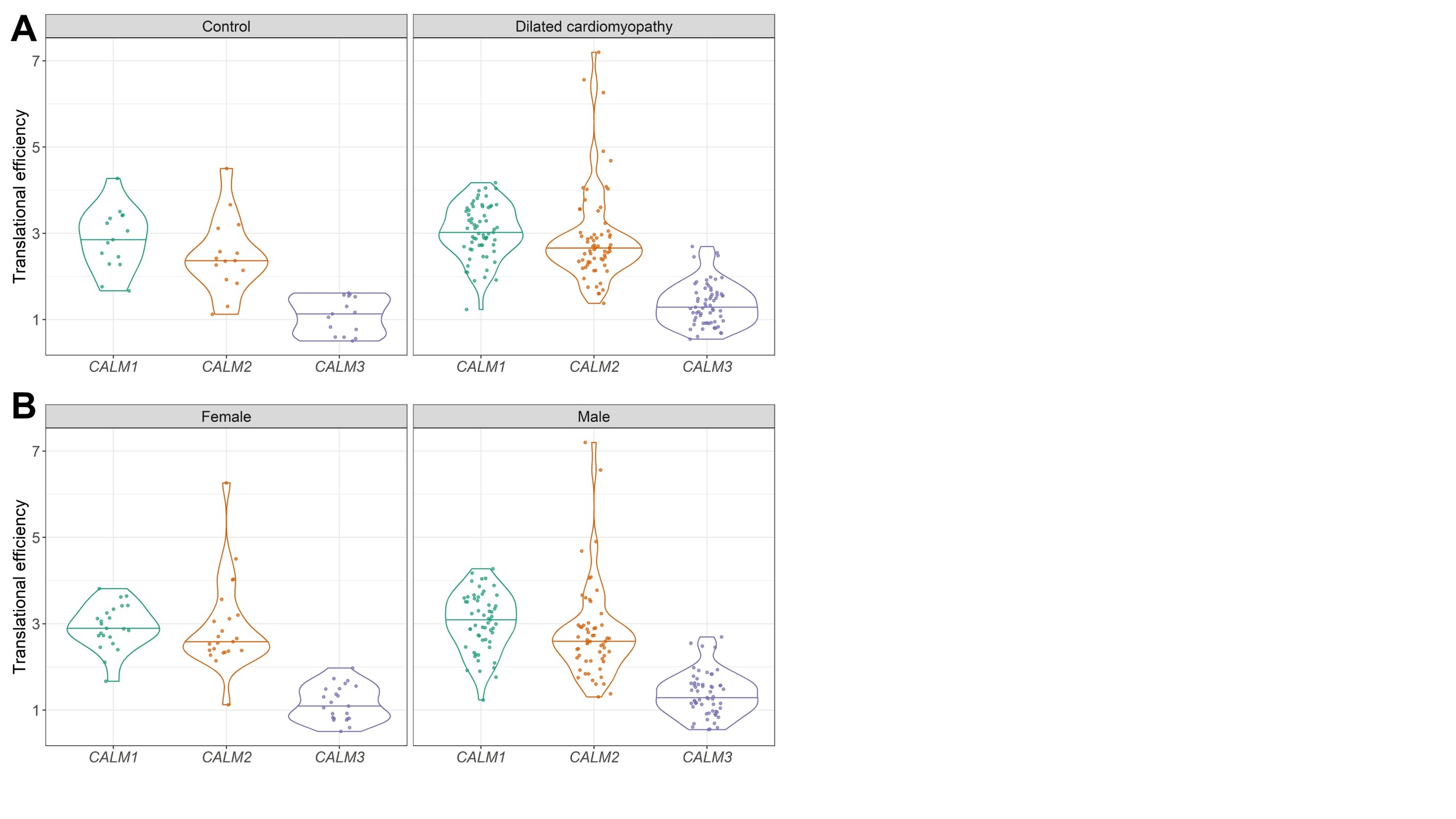 |
| --- |
| **Supplementary figure 4 \| Violin plots of translational efficiency per individual among *CALM1*, *CALM2*, and *CALM3*.**  A) Translational efficiency for controls (n = 15) and dilated cardiomyopathy patients (n = 65). Wilcoxon signed rank test among controls: *CALM1* vs *CALM2*, *p* = 0.095; *CALM1* vs *CALM3*, *p* = 6.1×10^-5^; *CALM2* vs *CALM3, p* = 1.2×10^-4^. Wilcoxon signed rank test among dilated cardiomyopathy patients: *CALM1* vs *CALM2*, *p* = 0.015; *CALM1* vs *CALM3*, *p* = 3.0×10^-12^; *CALM2* vs *CALM3, p* = 6.8×10^-12^. B) Translational efficiency for females (n = 23) and males (n = 57). Wilcoxon signed rank test among females: *CALM1* vs *CALM2*, *p* = 0.35; *CALM1* vs *CALM3*, *p* = 2.4×10^-7^; *CALM2* vs *CALM3, p* = 2.4×10^-7^. Wilcoxon signed rank test among males: *CALM1* vs *CALM2*, *p* = 4.3×10^-3^; *CALM1* vs *CALM3*, *p* = 6.5×10^-11^; *CALM2* vs *CALM3, p* = 2.4×10^-10^. Controls and dilated cardiomyopathy patients were present among both sexes. |
